# Tag SNPs of long non-coding RNA TINCR affect the genetic susceptibility to gastric cancer in a Chinese population

**DOI:** 10.18632/oncotarget.13513

**Published:** 2015-11-23

**Authors:** Xiang Ma, Chi Huang, Dakui Luo, Younan Wang, Ran Tang, Xiangkun Huan, Yi Zhu, Zekuan Xu, Ping Liu, Li Yang

**Affiliations:** ^1^ Department of General Surgery, The First Affiliated Hospital of Nanjing Medical University, Nanjing, China; ^2^ Jiangsu Province Academy of Clinical Medicine, Institute of Tumor Biology, Nanjing, China; ^3^ Department of Oncology, The First Affiliated Hospital of Nanjing Medical University, Nanjing, China

**Keywords:** gastric cancer, TINCR, polymorphism, genotype

## Abstract

Tissue differentiation-inducing non-protein coding RNA (TINCR) is required for normal epidermal differentiation. TINCR is also strongly overexpressed in human gastric cancer (GC) and contributes to carcinogenesis and tumor progression. However, the association between TINCR polymorphisms and the risk of any diseases, such as GC, remains unknown. In the present study, the tag single nucleotide polymorphisms rs8113645, rs2288947, rs8105637, and rs12610531 were analyzed in 602 patients with GC and 602 age- and sex-matched controls. Polymorphisms were genotyped using TaqMan technology. Carriers of variant rs8113645 and rs2288947 alleles indicated reduced risks of GC (*p* = 0.003 and 0.037, respectively). A allele genotypes of rs8113645 and G allele genotypes of rs2288947 (rs8113645 GA and AA; rs2288947 AG and GG) were also significantly associated with decreased GC risk (*p* < 0.05). Stratification analysis displayed that the correlations between GC risk and variant genotypes of both rs8113645 and rs2288947were more evident in younger individuals, men, nonsmokers, and individuals from rural areas. We also demonstrated that rs8113645 GA+AA genotype carriers had lower TINCR mRNA expression levels compared with common genotype in both normal and GC tissues (*p* < 0.05). These results suggest that long non-coding RNA TINCR polymorphisms may be implicated in GC development.

## INTRODUCTION

Gastric cancer (GC) is one of the most familiar cancers globally and leading to the second cause of cancer-associated mortality [1]. However, despite accumulating evidence indicating the involvement of multiple gene–environment interactions, the precise mechanisms of GC development remain poorly understood [2]. Our previous epidemiological studies of genetic variation had recognized genetic polymorphism as a crucial factor in the development of GC [3–6].

Long non-coding RNAs (lncRNAs) are non-coding transcripts containing more than 200 nucleotides. lncRNAs have received much attention in relation to their diverse biological functions [7]. Many biological processes, such as genomic imprinting, regulation of gene expression, dosage compensation, compartmentalization and nuclear organization, and nuclear–cytoplasmic trafficking are regulated by lncRNAs [8–10]. Furthermore, several studies have demonstrated dysregulation of many lncRNAs in various human diseases and disorders, including GC [11–14].

Tissue differentiation-inducing non-protein coding RNA (TINCR), named PLAC2 as well, is an lncRNA located on chromosome 19 in humans, producing a 3.7-kilobase transcript that is induced more than 150-fold during the process of epidermal differentiation. A previous study demonstrated the involvement of TINCR in epidermal differentiation in relation to normal induction of core mediatory protein of epidermal differentiation, and also in inducing genes involved in forming the cellular structures responsible for mediating differentiation-associated epidermal barrier formation [15]. Kretz et al. primarily verified that TINCR could interact with the double-stranded RNA-binding protein staufen1 (STAU1) and mediated the stabilization of differentiated mRNA [16]. Except for its function in localization of RNA to different subcellular compartments, STAU1 has also been implicated in RNA stability and acceleration of mRNA translation in mammalian tissues and cells [17–19].

Compared with normal adjacent gastric epithelium tissues, TINCR expression levels were aberrantly up-regulated in GC tissues, indicating a novel mechanism for TINCR in tumor development. The aberrant expression was associated with TNM stage and invasion depth of gastric tumors, and was also related to more dismal outcomes in patients with GC [20]. However, the correlation between TINCR genetic variants and the susceptibility of GC remains undetermined.

In view of the role of TINCR in carcinogenesis and tumor formation, we proposed the hypothesis that single nucleotide polymorphisms (SNPs) in the lncRNA TINCR may affect genetic susceptibility to GC. We therefore carried out a study of selected tag SNPs (rs8113645, rs2288947, rs8105637, rs12610531) across the whole TINCR locus to test the association between functional TINCR genotypes and GC risk in a Chinese population.

## RESULTS

### Demographic information

As shown in Table 1. The present study included 602 patients with GC and 602 age- and sex-matched cancer-free controls. No statistically significant difference was found between the two groups according to gender, age, hypertension, diabetes mellitus, and residence. However, the ratio of smokers was remarkably higher among GC cases compared with controls (21.3% vs. 15.6%, *p* = 0.012).

**Table 1:** Demographic information

Characteristics	Cases (n = 602)	Controls (n = 602)	*P* value
Age (y)*	60.6±10.7	59.5±12.9	0.087
Gender, (n (%))			
Male	438 (72.8)	409 (67.9)	0.067
Hypertension, (n (%))			
Yes	172 (28.6)	181 (30.1)	0.569
Yes	61(10.1)	76(12.6)	0.173
Smoking, (n (%))			
Smokers	128(21.3)	94 (15.6)	**0.012**
Residence, (n (%))			
Urban	244 (47.4)	271 (52.0)	0.116
Tumor differentiation (n (%))			
Depth of tumor infiltration (n (%))			
Lymph node metastasis (n (%))			
Localization (n (%))			
Noncardia	336(55.8)		

* Median (25th-75th percentiles).

### Association between TINCR SNPs and GC risk

The allele and genotype frequencies of the lncRNA TINCR SNPs and their associations with GC risk are summed up in Table 2. All the four SNPs did not depart from the Hardy–Weinberg equilibrium in the healthy controls in both cases and controls (*p* > 0.05). The frequency of the rs8113645 A allele was obviously increased in controls compared with GC subjects (*p* = 0.003). Taking GG genotype as a comparison, the variant GA, AA, and GA+AA genotypes were significantly associated with a decreased risk of GC (*p* = 0.033, 0.036, and 0.010 respectively).

**Table 2:** Association between TINCR gene polymorphisms and risk of gastric cancer

genotype	Cases N (%)	Controls N (%)	Crude OR^a^ (95% CI^b^)	*P* value	Adjusted OR (95% CI)*	*P* value
rs8113645						
GA	121 (20.1)	150(24.9)	**0.74 (0.56-0.97)**	**0.031**	**0.74 (0.56-0.98)**	**0.033**
AA	8 (1.3)	18(3.0)	**0.04(0.18-0.95)**	**0.037**	**0.040(0.17-0.94)**	**0.036**
GA + AA	129 (21.4)	168(27.9)	**0.71 (0.54-0.92)**	**0.009**	**0.71 (0.54-0.92)**	**0.010**
rs2288947						
AG	227 (37.7)	257 (42.7)	**0.76 (0.60-0.96)**	**0.023**	**0.76 (0.60-0.97)**	**0.026**
GG	52 (8.6)	68 (11.3)	**0.66(0.44-0.97)**	**0.036**	**0.63(0.42-0.95)**	**0.026**
AG + GG	279(46.3)	325 (54.0)	**0.74 (0.59-0.92)**	**0.008**	**0.74 (0.59-0.92)**	**0.008**
rs12610531						
AG	305 (50.7)	318(52.8)	0.93 (0.71-1.22)	0.594	0.93 (0.71-1.23)	0.621
GG	142 (23.5)	134(22.3)	1.02(0.74-1.41)	0.913	1.01(0.73-1.41)	0.947
AG + GG	446 (74.2)	452(75.1)	0.96 (0.74-1.24)	0.728	0.96 (0.74-1.24)	0.735
rs8105637						
AG	259 (43.0)	247 (41.0)	1.09 (0.86-1.38)	0.486	1.07 (0.84-1.36)	0.591
AA	50 (8.3)	51 (8.5)	1.02(0.67-1.55)	0.937	1.05(0.68-1.61)	0.831
AG + GG	339 (51.3)	298 (49.5)	1.08 (0.86-1.35)	0.526	1.06 (0.84-1.34)	0.609
HWE	0.493	0.934				

Abbreviations: *Adjusted for age, sex, smoking status, residence, hypertension, and diabetes.

^a^OR, odds ratio;

^b^CI, confidence interval

^c^HWE, Hardy–Weinberg expectations

For rs2288947, the variant AG, GG, and GG+AG genotypes and G allele were also correlated with a remarkably reduced risk of GC (*p* = 0.026, 0.026, 0.008 and 0.037 respectively), compared with the AA genotype and A allele. However, we didn't detect any significant associations between the genotypes of the other two TINCR SNPs and GC risk.

### Functional relevance of rs8113645 and rs2288947 to TINCR expression

**Figure 1:**
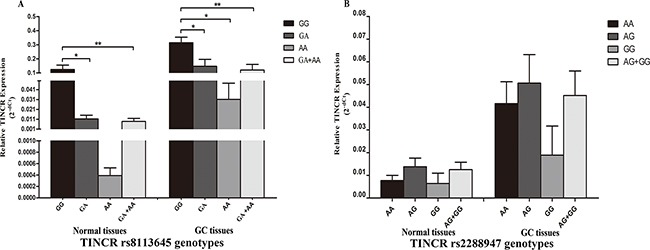
Correlation between rs8113645 and rs2288947 genotypes and expression of TINCR mRNA **A**. Genotype-phenotype correlation for rs8113645 and relative expression levels of TINCR mRNA. Relative TINCR mRNA expression levels were significantly lower for the GA (0.011 ± 0.004) and GA+AA genotypes (0.009 ± 0.003) than the GG genotype (0.124 ± 0.003) in normal tissues (*P < 0.05; **P < 0.01). In gastric cancer tissues, relative TINCR mRNA expression levels were significantly lower for the GA (0.147 ± 0.0049), AA (0.031 ± 0.016) and GA+AA genotypes (0.121 ± 0.039) than the GG genotype (0.314 ± 0.039) (*P < 0.05; **P < 0.01). **B**. Genotype-phenotype correlation for rs2288947 and relative expression levels of TINCR mRNA. Relative TINCR mRNA expression levels were similar among the three groups with rs2288947 AA, AG and GG genotypes in both normal and gastric cancer tissues.

We characterized the expression of TINCR and correlated it with the different genotypes of the rs8113645 and rs2288947 SNPs in 66 paired GC and non-GC samples, using real-time–PCR. For rs8113645, the 66 samples included 44 GG, 17 GA, and five AA genotypes. As shown in Figure 1A, subjects carry rs8113645 GA genotype had significantly lower TINCR mRNA levels (mean ± standard error) compared with those with the GG genotype, in both normal and cancerous gastric tissues (normal tissues: 0.011 ± 0.004 VS. 0.124 ± 0.003, *p* < 0.05; GC tissues: 0.147 ± 0.0049 VS. 0.314 ± 0.039, *p* < 0.05). Analogic results were discovered when the TINCR mRNA levels were compared in relation to rs8113645 GG and GA+AA genotypes (normal tissues: 0.009 ± 0.003 VS. 0.124 ± 0.003, *p* < 0.05; GC tissues: 0.121 ± 0.039 VS. 0.314 ± 0.039, *p* < 0.01). However, TINCR mRNA levels were significantly lower in rs8113645 AA compared with rs8113645 GG genotype individuals only in GC tissues (GC tissues: 0.031 ± 0.016 VS. 0.314 ± 0.039, *p* < 0.01).

The frequency distributions of rs2288947 genotypes AA, AG, and GG were 37, 24, and five, respectively (Figure 1B). Relative TINCR mRNA expression levels were similar in all three groups in both normal and GC tissues (normal tissues: AA (0.008 ± 0.002), AG (0.014 ± 0.004), and GG (0.006 ± 0.005); GC tissues: AA (0.042 ± 0.009), AG (0.051 ± 0.013), and GG (0.019 ± 0.013)].

### Stratification analysis of SNPs and GC risk

We investigated the interactions between the meaningful SNPs and potential factors including median age (60 years), gender, smoking situation, and residence. The results are summed up in Table 3. The decreased risks associated with the variant genotypes of rs8113645 and rs2288947 are more evident in individuals aged <60 years (*p* = 0.016 and 0.027 respectively), men (*p* = 0.001 and 5.3×10^-5^ respectively), non-smokers (*p* = 0.032 and 0.035 respectively), and individuals living in rural areas (*p* = 0.019 and 0.042 respectively). However, the associations were not significant in subjects aged ≥60 years, women, smokers, and individuals from urban areas.

**Table 3:** Stratified analyses for TINCR genotypes in cases and controls

Variable	n GA+AA (%)/n GG (%) for rs8113645		Allelic odds ratios and 95% confidence intervals for rs8113645		n AG+GG (%)/ n AA (%) for rs2288947		Allelic odds ratios and 95% confidence intervals for rs2288947	
	Cases	Controls	Adjusted OR (95% Cl)*	*P* value	Cases	Controls	Adjusted OR (95% Cl)*	*P* value
**Age (y), median**								
**≥ 60**	72(12.0)/247(41.0)	75(12.5)/206(34.2)	0.77 (0.52-1.12)	0.172	151(25.1)/168(27.9)	152(25.2)/129(21.4)	0.74 (0.53-1.03)	0.075
**<60**	58(9.60)/225(37.4)	93(15.4)/228(37.9)	**0.63(0.43-0.92)**	**0.016**	128(21,3)/l 55(25.7)	173(28.7)/148(24.6)	**0.69 (0.50-0.96)**	**0.027**
**Sex**								
**Females**	43(7.10)/121(20.1)	46(7.6)/147(24.4)	1.17(0.72-1.90)	0.532	92(15.3)/72(12.0)	91(15.1)/ 73(12.1)	1.39(0.91-2.12)	0.133
**Males**	87(14.5)/351(58.3)	122(20.3)/287(47.7)	**0.58(0.42-0.80)**	0.001	187(31.1)/251(41.7)	234(38.9)/204(33.9)	**0.57 (0.43-0.75)**	5.3*10^−5^
**Smoking Status**								
**Smokers**	26(4.3)/102(16.9)	27(4.5)/67(11.1)	0.63 (0.33-1.20)	0.161	60(10.0)/68(11.3)	64(10.6)/64(10.6)	0.65 (0.38-1.13)	0.129
**Nonsmokers**	104(17.3)/370(61.5)	141(23.4)/367(61.0)	**0.73 (0.54-0.97)**	**0.032**	219(36.4)/255(42.4)	261(43.4)/213(35.4)	**0.76 (0.59-0.98)**	**0.035**
**Residence**								
**Rural**	75(12.5)/283(47.0)	94(15.6)/237(39.4)	**0.65(0.46-0.93)**	**0.019**	164(27.2)/194(32.2)	192(31.9)/166(27.6)	**0.73 (0.54-0.99)**	**0.042**
**Urban**	55(9.1)/189(31.4)	74(12.3)/197(32.7)	0.78 (0.52-1.17)	0.229	115(19.1)/129(21.4)	133(22.1)/l 11(18.4)	0.75 (0.53-1.07)	0.111

^*^ Adjusted for age, sex, smoking status, residence, hypertension, and diabetes.

We also explored the interactions between the mutated genotypes and clinicopathological characteristics in GC patients (Table 4). No significant association was discovered between the SNPs and clinicopathological characteristics, such as tumor infiltrating depth, differentiation grade, lymph node metastasis, or position of the primary cancer.

**Table 4:** Associations between variant TINCR genotypes and clinicopathologic characteristics of gastric cancer

Variable	GA+AA, GG for rs8113645		Allelic odds ratios and 95% confidence intervals for rs8113645		AG+GG, AA for rs2288947		Allelic odds ratios and 95% confidence intervals for rs2288947	
	CT+TT, n	CC, n	Adjusted OR(95%CI)*	*P* value	AG+GG, n	AA, n	Adjusted OR (95% CI)*	*P* value
Tumor differentiation								
Moderate	30	103	0.93 (0.29-2.92)	0.896	64	69	0.90 (0.35-2.30)	0.825
Poor	95	351	1.01 (0.36-2.83)	0.982	204	242	0.97 (0.41-2.29)	0.943
Depth of tumor infiltration								
T2	11	57	0.87(0.37-2.07)	0.752	34	34	1.12(0.57-2.22)	0.746
T3	69	200	1.43 (0.79-2.58)	0.240	118	151	0.86 (0.53-1.40)	0.547
T4	32	143	0.90 (0.46-1.76)	0.750	84	91	1.03 (0.61-1.75)	0.905
Lymph node metastasis								
Positive	84	322	0.85 (0.56-1.28)	0.431	184	222	0.87(0.62-1.23)	0.428
Localization								
Noncardia	78	258	1.22(0.82-1.82)	0.327	167	169	1.32 (0.95-1.84)	0.094

* Adjusted for age, sex, smoking status, residence, hypertension, and diabetes.

## DISCUSSION

To be sure, this study provides the first evidence for an association between lncRNA TINCR polymorphisms and GC risk in a Chinese Han population. Variant genotypes of both rs8113645 (GA/AA) and rs2288947 (AG/GG) were associated with significantly reduced risks of GC. Furthermore, TINCR tissue expression levels differed according to rs8113645 genotypes, with lower levels in carriers of the A allele. As a novel class of noncoding RNAs, lncRNAs have attracted increasing attention, especially in cancer research [21]. Although the specific functions of most lncRNAs remain unknown, recent studies have been initiated to elucidate their mechanisms in cancer development and progression [22]. Furthermore, studies have suggested that polymorphisms in lncRNAs may affect their expression and subsequently contribute to GC susceptibility [4, 23, 24].

TINCR is an lncRNA that is highly induced during epidermal differentiation [16]. Xu et al. reported that TINCR expression was aberrantly overexpressed in GC tissues in comparison with corresponding noncancerous tissues, and verified that TINCR overexpression was induced by nuclear transcription factor SP1. TINCR silencing in SGC7901 and BGC823 cell lines inhibited colony formation, cell proliferation, carcinogenicity, and apoptosis promotion, yet the overexpression of TINCR promoted cell growth. They also showed that TINCR could interact with STAU1 protein and affect KLF2 mRNA expression and stability, thereby affecting the transcription and expression of the KLF2-regulated cyclin-dependent kinase genes CDKN1A/P21 and CDKN2B/P15 and impacting on the proliferation and apoptosis of GC cells [20]. These results demonstrated that TINCR may act as an oncogene in GC. The contradictory role of TINCR in inducing epidermal differentiation and GC development could be explained that lncRNAs may regulate biological processes by various mechanisms, such as DNA methylation, chromatin remodeling, transcriptional regulation, post-transcriptional regulation, transformation of stemness features and competitive endogenous RNAs [25–29]. A specific lncRNA could exert diverse roles under different cellular circumstances. TINCR manages human epidermal differentiation by a post-transcriptional mechanism. It can interact with a sequence of differentiation mRNAs at post-transcriptional level. On the contrary, TINCR promotes the proliferation of gastric cancer cells. Although it sounds to be paradoxical, some rational explains could be applied to clarify the issue. It is demonstrated that TINCR could interact with STAU1 protein and alter KLF2 mRNA stability. It is deemed to be that TINCR develop its regulatory mechanism in a transcriptional level. The reverse outcomes may attribute to different mechanisms. Besides that, in a particular biological condition, the various regulatory mechanisms of the identical lncRNA could appear simultaneously. Some weak effects could be overwhelmed by the dominant mechanisms, resulting in inconsistent biological phenotypes.

In the current study, we identified an association between the rs8113645 A allele and decreased TINCR expression in normal and GC tissues. rs8113645 G>A mutations may thus decrease the carcinogenic effect of TINCR, indicating a biological basis for the association. The exactly mechanisms underlying TINCR SNPs involved in decreasing risk for GC are still undetermined. It is reported that some lncRNAs acting as competing endogenous RNAs (ceRNA) function as miRNA sponges which sequester miRNA to regulate other transcripts expression level by sharing common miRNA response elements [30, 31]. We speculate that variants in miRNA binding site could result in gain and lose of function of miRNA-lncRNA interaction ultimately affect other miRNA targeted mRNA expression [32]. As predicted by http://bioinfo.life.hust.edu.cn/lncRNASNP/, the conversion of rs8113645 G>A of TINCR may create hsa-miR-30c-1-3p, hsa-miR-30c-2-3p, hsa-miR-3192-5p, and hsa-miR-6788-5p, and destroy hsa-miR-204-3p and hsa-miR-4646-5p micro RNA (miRNA)-binding sites on TINCR, leading to gain or loss of function of miRNA–lncRNA interactions. Similarly, the rs2288947 A>G polymorphism may cause miRNA–lncRNA gain by binding hsa-miR-665 or hsa-miR-6840-3p, and miRNA–lncRNA loss by binding hsa-miR-1247-3p. Accumulating evidence suggests that miRNAs can directly regulated lncRNAs [33], and the effect of SNPs on miRNA-binding sites may thus change the structure, and consequent function, of TINCR. What's more, TINCR SNPs may have a genotype-phenotype correlation on its nearby genes ultimately decreased GC risk [31]. Further studies exploring the precise mechanisms involved in these interactions are awaited.

Stratified analysis in the present study revealed that both rs8113645 and rs2288947 polymorphisms were associated with reduced risks of GC in patients aged <60 years, men, non-smokers, and individuals living in rural areas, but conversely not in those aged ≥60 years, women, smokers, and people from urban areas. In general, GC was more common in men and older subjects. A previous study reported that old individuals are susceptible to environmental carcinogens and their immune system are vulnerable [34], thus the rs8113645 and rs2288947 variant genotypes effects may be tended to be age-specific. Another study in a Chinese population indicated that non-cardia GC was more usual in men than women, with a ratio of about 2:1, while the ratio of male to female was 4.1:1 in gastric cardia cancer [35]. These findings suggest that rs8113645 and rs2288947 polymorphisms play important roles in women with GC.

Analyses stratified according to smoking situation and residence revealed significant protective effects of the rs8113645 A allele and rs2288947 G allele in non-smokers and people from rural areas, but not in smokers and those from urban areas. Tobacco smoke is a confirmed independent risk factor for GC [36], and it is likely that any association between polymorphisms and GC risk may be concealed by the overwhelming effect of collective exposure to tobacco carcinogens in smokers, leading to a more significant association among non-smokers [37]. Environmental factors may also account for the different effects of polymorphisms in individuals from rural and urban areas, with genetic differences having a stronger effect under conditions of low environmental pollution [2, 38]. However, more evidence is needed to confirm these conclusions.

This case-control study had several limitations. Firstly, the subjects were consecutively enrolled from a single hospital, during the same period, selection bias is inevitable. However, the distribution of genotype in the healthy control subjects met standards for Hardy–Weinberg expectations. Secondly, the sample size is relatively inadequate, which may have limited the analysis statistical power. Thirdly, although *Helicobacter pylori* is an independent pathogenic factor for GC, we did not investigate this variable because it would have been unethical to carry out a *H. pylori* test for every participant, particularly in healthy controls. Fourthly, a high-salt diet, alcohol consumption, chronic gastric ulcer, and family history of GC are crucial factors in gastric carcinogenesis, but we failed to perform gene–environment interaction analyses for these because of limitations in the collected data. Fifthly, in terms of screening for candidate SNPs in present work, selection based on genotype data for Han Chinese in Beijing from the HapMap database remains a core criterion. However, the locations of the SNPs and some significant SNPs also should be taken into consideration. Lastly, the study was designed on the condition of Chinese population, and the conclusions should thus be extended to other ethnic groups with caution.

In conclusion, this study indicated that two SNPs of the lncRNA TINCR (rs8113645 G>A and rs2288947 A>G) were significantly associated with decreased GC susceptibility, and the rs8113645 G>A SNP may reduce susceptibility to GC by decreasing gene expression levels. Our results pose new direction for TINCR variants in GC carcinogenesis, and further prospective studies based on larger sample size are required for verifying these initial findings.

## MATERIALS AND METHODS

### Study subjects

This study was authorized by the ethics committee of First Affiliated Hospital of Nanjing Medical University (Nanjing, China). Before each subject was recruited, a written informed consent was obtained. The present hospital-based, case-control study enrolled 602 GC patients and 602 cancer-free controls. All the subjects were genetically irrelevant and dwelled in Jiangsu province or ambient area. All GC cases were randomly enrolled from the First Affiliated Hospital of Nanjing Medical University from 2009 to 2015, and all diagnoses were confirmed by examination of gastroscopic biopsy or surgical specimens. The age- (±5 years) and sex-matched controls were randomly enrolled at the same hospital during the same time period, and were confirmed to have no current or previous signs of cancer. Individual with additional recurrent malignancies, genetic diseases, who received non-self blood transfusions, or who received chemotherapy or radiotherapy were excluded from this study. Basic information including age, sex, smoking conditions, urban or rural residence, hypertension, diabetes, and clinical information (tumor site, histological type, clinical tumor node metastasis by UICC/AJCC criteria of TNM stage) were collected by questionnaires or from medical records. A smoker was defined as an individual who smoked ≥10 cigarettes per day formerly or currently for no less than 2 years.

### SNP selection

We selected Tag SNPs basing on genotype information for Han Chinese in Beijing from the HapMap database (HapMap Data Rel 27, Phase II+III, Feb09, on NCBI B36 assembly, dbSNP b126). Finally, four tag SNPs (rs8113645, rs2288947, rs8105637, rs12610531) were selected summarizing all the common SNPs (minor allele frequency >0.05) located at the chromosome locus transcribed into TINCR. Pairwise option of Haploview 4.2 software (Cambridge, MA, USA) was used to conduct the selection, and the threshold for further investigation was set as r^2^ > 0.8[39].

### Genotyping

Genomic DNA was extracted from 2 ml of venous blood following standard protocols as presented previously [34]. The purity and concentration were determined using a NanoDrop Spectrophotometer (ND-1000) and all DNA samples were diluted to a particular concentration of 10ng/μl before analysis. The selected SNPs (rs8113645, rs2288947, rs8105637, rs12610531) were genotyped using TaqMan allelic discrimination methods with an ABI StepOnePlus real-time PCR system, according to the official instructions (Applied Biosystems Foster City, CA, USA). PCR was performed in a 10-μl reaction volume consisting of 10 ng of DNA template, 5 μl 2× TaqMan Universal PCR master mixes, 0.5 μl of primer pairs, 0.25 μl TaqMan minor groove-binding probes, and 2.5 μl double distilled water. The sequences of the primers and probes are detailed in Supplementary Table S1. Amplification was conducted under the following reactive conditions: 95°C for 10 min, followed by 40 cycles at 95°C for 15s and 60°C for 1min. The average call rates for each SNP were >98%; about 10% of samples were chosen randomly for repeat assays, and concordance rate was 100% in the final. The direct DNA sequencing technique was used to verify the exact genotypes of the three polymorphisms (except the rs8105637, for the technical reasons) (Supplementary Figure S1), and the results were 100% concordant.

### Real-time analyses of lncRNA TINCR

Total RNA was extracted from 66 paired cancerous and normal gastric tissues with TRIzol reagent (Invitrogen, Carlsbad, CA, USA), and each isolated RNA sample was converted to cDNA with Primescript RT Reagent (Takara, Otsu, Japan). Relative gene expression levels of TINCR were carried out using glyceraldehyde 3-phosphate dehydrogenase (GAPDH) as an internal reference gene, and High ROX Premixed SYBR-Green Master Mix (Vazyme, Nanjing, China) with the ABI StepOnePlus RealTime PCR System, in triplicate. The primers of TINCR used for quantitative real-time PCR were delineated as follows: forward 5′-CCTTCCCATCTGTTCTCCCTTCC-3′ and reverse 5′-CTGTATCTAGTTCCAAGCTGGGTGAT-3′. GAPDH was used as an internal control and amplified with the following primers: 5′-CGACCACTTTGTCAAGCTCA-3′ and reverse 5′-AGGGGTCTACATGGCAACTG-3′. Each amplification reaction was finished in a total volume of 10 μl containing 0.2 μl primers, 5 μl Master mix and 100 ng of the cDNA. Reaction conditions were set as follows: 95°C for 30 s, 95°C for 5 s and 60°C for 30 s for 40 cycles.

### Statistical analysis

SPSS 22.0 software was used to perform all statistical analyses, with a significance level of *p* < 0.05. All tests were two-sided. Differences in demographic variables were distinguished using Student's *t*-tests and χ^2^ tests. Hardy–Weinberg equilibrium was evaluated for controls using the goodness-of-χ^2^ test. Quantitative variables that departed from a normal distribution were summarized as medians and analyzed using Mann–Whitney rank-sum tests. The associations between gene variants/genotypes and GC risk were computed as ORs and 95% CIs. Crude ORs were computed with the Woolf approximation method, and adjusted ORs were calculated by multivariate analysis with unconditional logistic regression, with adjustment for age, hypertension, gender, smoking condition, diabetes, and residence. The relative expression levels of TINCR in all samples were worked out using 2^−Δct^ method, compared with the levels of GAPDH. The associations between the expression levels of TINCR and TINCR polymorphisms were evaluated by one-way ANOVA, post-hoc test.

## SUPPLEMENTARY FIGURE AND TABLE


