# Supplementary material for: Tag SNPs of long non-coding RNA TINCR affect the genetic susceptibility to gastric cancer in a Chinese population
Source: Oncotarget. 2015 Nov 23;7(52):87114–23. doi: 10.18632/oncotarget.13513 (PMC5349975; doi:10.18632/oncotarget.13513)
Supplement: Supplementary file 1 [file oncotarget-07-87114-s001.pdf]

Tag SNPs of long non-coding RNA TINCR affect the genetic susceptibility to gastric cancer in a Chinese population

SUPPLEMENTARY FIGURE AND TABLE

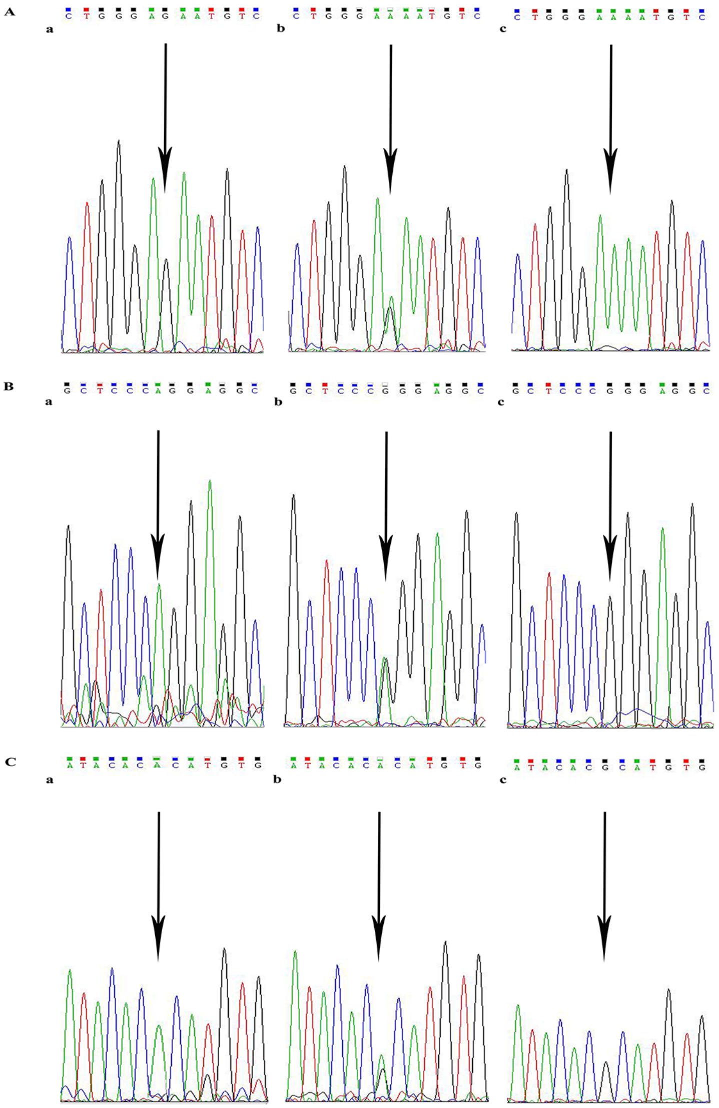

Supplementary Figure S1: Direct sequencing results for the tag SNPs of long non-coding RNA TINCR. The polymorphisms were detected by TaqMan technology and confirmed by direct polymerase chain reaction sequencing (except the rs8105637, for the technical reasons). The single base indicated by a black arrowhead is the site of the small nucleotide polymorphism. **A.** Representative DNA sequencing results of rs8113645: (a) GG, (b) GA and (c) AA genotypes; **B.** Representative DNA sequencing results of rs2288947: (a) AA, (b) AG and (c) GG genotypes; **C.** Representative DNA sequencing results of rs12610531: (a) AA, (b) AG and (c) GG genotypes.

Supplementary Table S1: Information of primers and probes

SNPs	Primer sequence(5'-3')	Probe sequence
rs8113645 G>A	F-CCAGGGCTGTTCACTGCACAAAGG	T:FAM-CCCAGGACATTTTCCCAGCCACACC-MGB
	R-AGCCAGAGGGCATCGCAAGAAG	C:HEX-CCCAGGACATTCTCCCAGCCACACC-MGB
rs2288947 A>G	F-AGTGTATGGAGGGGGATGATGGT	T:FAM-CTCCTGCCTCCTGGGAGCCTAGATC-MGB
	R-GAATAGGCTGGGGTAGAGGGCA	C:HEX-CTCCTGCCTCCCGGGAGCCTAGATC-MGB
rs8105637 A>G	F-GACAGAGCTTCAGAGACAGAGAAAA	A:FAM-CAGAGACACCAGAGAG-MGB
	R-TCTCTGTTATTTTGGTCTCTGCTCT	G: HEX-CAGAGACACCGGAGAG-MGB
rs12610531 A>G	F-GGCATGGAGTAAGGACCAAGGAA	T:FAM-GGGCCACATGTGTGTATCTAGAGCCTG-MGB
	R-AGGCCCAAGGAGGTTGTCAGG	C:HEX-GGGCCACATGCGTGTATCTAGAGCCTG-MGB
